# P-756. Primary Mycobacterium Infections presenting in patients of Rheumatic diseases - A Case Series

**DOI:** 10.1093/ofid/ofae631.951

**Published:** 2025-01-29

**Authors:** Aalesh Shah, Yug Patel, Dhaiwat Shukla

**Affiliations:** Smt. NHL Municipal Medical College, Ahmedabad, Gujarat, India; Smt. NHL Municipal Medical College, Ahmedabad, Gujarat, India; V.S. General Hospital, Ahmedabad, Gujarat, India

## Abstract

**Background:**

We present a study about patients with rheumatic diseases who were diagnosed for mycobacterium infections in their follow-up visit. The role of side effects of immunosuppressants have been established. However, their association with mycobacteria in endemic countries like India is yet to be understood well.

ABDOMINAL CT - CASE 1 (TUBERCULAR PERITONITIS)

**Figure:**
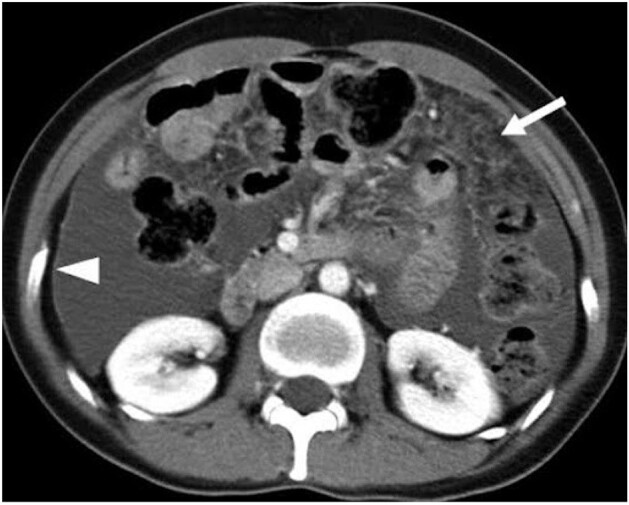

Moderate to gross ascites with thickened enhancing parietal peritoneum, significant with caking and abdominal lymphadenopathy hence pointing towards Tubercular Peritonitis.

**Methods:**

Consent was obtained from the patients to use their data for a case series. Relevant data related to the cases was collected, and organised.

HRCT THORX - CASE 3 (PULMONARY TB)

**Figure:**
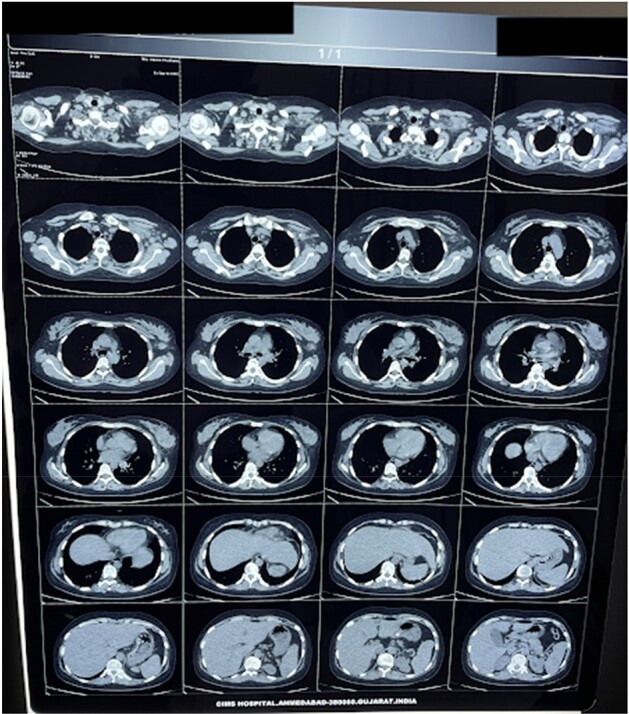

HRCT THORAX shows multiple, discrete, randomly distributed nodules in both the lungs, multiple enlarged lymph nodes in left axilla and in left anterior chest wall, some of which are confluent in nature and two calcifications in one of the lymph node.

**Results:**

First case is about a 54 yr-old male patient, known case of Ankylosing spondylitis treated with 9 doses of Anti-TNF alpha drug Adalimumab presented with severe abdominal pain, fever with chills, vomiting & diarrhea for which he underwent abdominal CT-scan which revealed thickened enhancing parietal peritoneum, significant with caking and abdominal lymphadenopathy hence pointing towards Tubercular Peritonitis. Second case is about Non-Tuberculous Mycobacteria(NTM) or atypical mycobacteria diagnosed in a patient with Ankylosing Spondylitis who had negative tests for primary TB. Third case about a 28 year-old female, known case of bilateral iridocyclitis and recurrent multiple medium-vessel vasculitic/throm2botic occlusions treated with mycophenolate-mofetil and cyclosporine-A, was started on adalimumab. Later she presented with fever for which pulmonary workup was done which revealed positive TB. Fourth case is of a patient having symptoms similar to Rheumatoid arthritis but not responsive to treatment which led to extensive workup and mycobacterium leprae was found as a culprit. Fifth case is of a 16 year-old male, known case of GPA who was refractory to cyclophosphamide was started on rituximab, after which he presented with complaints of fever with chills and rigors, and breathlessness on exertion which was followed by the diagnosis of miliary tuberculosis.

TB patients were given Anti-tubercular therapy(ATT) and Leprosy patient was given Multi-drug therapy(MDT) and all are now improving.

Leonine Facies - Case 4

**Figure:**
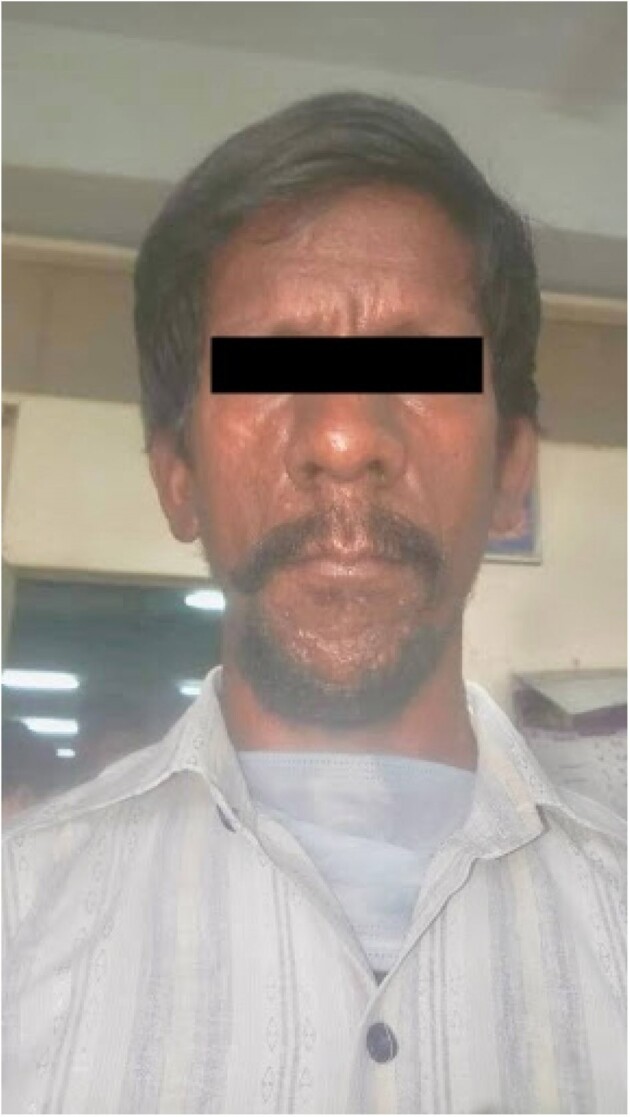

**Conclusion:**

• Our study shows that immunosuppressants can be considered to have even increased risk of primary mycobacterium infection.

• Leprosy can present with similar manifestations of RA (Rheumatoid Arthritis) so signs of suspicion need to be kept in mind and screening should be done in such cases.

Leprosy mimicking RA - Case 4

**Figure:**
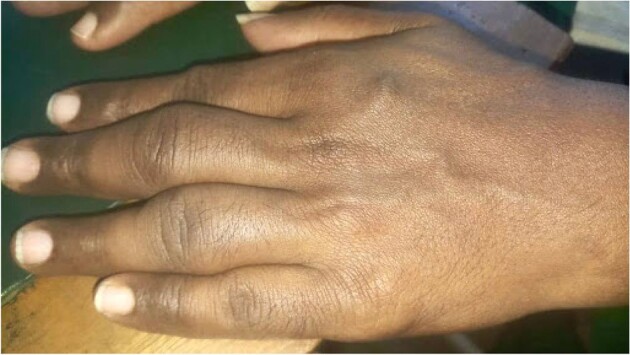

**Disclosures:**

**All Authors**: No reported disclosures

